# Potential Implications of a Type 1 Interferon Gene Signature on COVID-19 Severity and Chronic Inflammation in Sickle Cell Disease

**DOI:** 10.3389/fmed.2021.679030

**Published:** 2021-07-22

**Authors:** Emaan Madany, Derick Okwan-Duodu, Raisa Balbuena-Merle, Jeanne E. Hendrickson, David R. Gibb

**Affiliations:** ^1^Department of Pathology and Laboratory Medicine, Cedars-Sinai Medical Center, Los Angeles, CA, United States; ^2^Department of Laboratory Medicine, Yale New Haven Hospital, New Haven, CT, United States

**Keywords:** sickle cell disease, type 1 interferons, COVID-19, myxovirus resistance interferon stimulated genes, SARS-CoV-2

## Abstract

At the onset of the corona virus disease 19 (COVID-19) pandemic, there were concerns that patients with sickle cell disease (SCD) might be especially vulnerable to severe sequelae of SARS-CoV-2 infection. While two reports support this conclusion, multiple studies have reported unexpectedly favorable outcomes in patients with SCD. However, mechanisms explaining these disparate conclusions are lacking. Here, we review recent studies indicating that the majority of patients with SCD express elevated levels of anti-viral type 1 interferons (IFNα/β) and interferon stimulated genes, independent of COVID-19, during their baseline state of health. We also present our data from the pre-COVID-19 era, illustrating elevated expression of a well-characterized interferon stimulated gene in a cohort of patients with SCD, compared to race-matched controls. These type 1 interferons and interferon stimulated genes have the potential to contribute to the variable progression of COVID-19 and other viral infections in patients with SCD. While the majority of evidence supports a protective role, the role of IFNα/β in COVID-19 severity in the general population remains an area of current investigation. We conclude that type 1 interferon responses in patients with SCD may contribute to the variable COVID-19 responses reported in prior studies. Additional studies investigating the mechanisms underlying IFNα/β production and other clinical consequences of IFNα/β-mediated inflammation in SCD disease are warranted.

## Graphical Abstract

Hypothesis: Baseline type I interferon activity may contribute to variable COVID-19 progression in SCD. (Top) At early stages of SARS-CoV-2 infection, high baseline IFNα/β activity may contribute to the anti-viral response in patients with SCD. Recognition of damage-associated molecular patterns by pattern recognition receptors (PRRs) induces IFNα/β production. Heme released from hemolyzed sickle cells binds Toll-like receptor 4 (TLR4), which may induce IFNα/β in vascular endothelial cells. IFNα/β bind to the IFNα/β receptor (IFNAR) in neutrophils and other cells types, leading to production of MxA and other interferon-stimulated genes (ISGs). ISGs can directly inhibit viral replication and promote B cell production of neutralizing antibodies. The IFNα/β response is one of multiple responses, including production of IL-6, TNFα, and IL-1b, by innate and adaptive immune cells that have the potential to limit COVID-19 progression. (Bottom) In contrast, reduced or absent IFNα/β activity may increase susceptibility to viral infection, leading to airway epithelial cell death and COVID-19. Dashed lines indicate potentially connected pathways, while solid lines are supported by prior studies. 
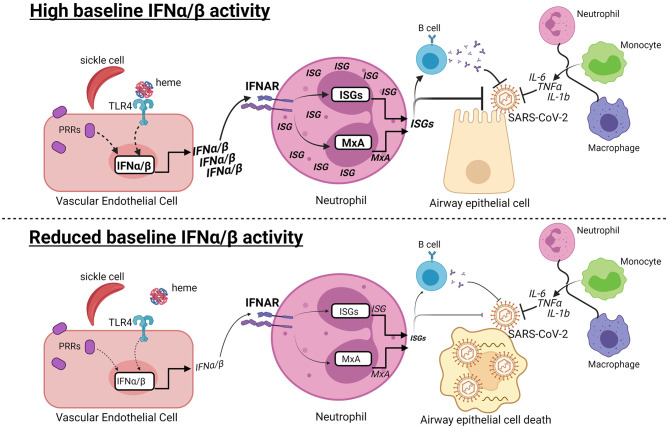

## Introduction

Certain comorbidities are associated with an increased severity of corona virus disease 19 (COVID-19) resulting from SARS-CoV-2 infection (1). Given that patients with sickle cell disease (SCD) have underlying chronic inflammation, significant cardiopulmonary dysfunction, and immune deficiency due in part to hyposplenism, concern exists regarding the severity of COVID-19 in patients with SCD (2). In March of 2020, the National Haemoglobinopathy Panel in the United Kingdom deemed that patients with SCD and other rare hemoglobinopathies are exceptionally vulnerable populations requiring strict self-isolation (3). In November, the Center for Disease Control added SCD to the list of comorbidities that may increase the severity of COVID-19 (https://www.cdc.gov/coronavirus/2019-ncov/need-extra-precautions/people-with-medical-conditions.html).

Multiple case series and reports have described the disease course of patients with SCD and COVID-19. One common finding is that COVID-19, like H1N1 influenza and other respiratory infections, can trigger acute chest syndrome and vaso-occlusive crises (4, 5). However, it is unclear whether patients with SCD have a more or less severe COVID-19 disease course than those without SCD. Minniti et al. reported that pre-existing co-morbidities, including renal and cardiopulmonary disease, are associated with poor COVID-19 outcomes in patients with SCD (6). In addition, Singh et al. found an increase in hospitalizations and pneumonia, but no increase in mortality, when comparing patients with SCD to race-matched controls with comorbidities (7). In contrast, smaller studies have reported favorable outcomes in patients with SCD (4, 8–11). As reviewed by Sahu et al., most SARS-CoV-2 infected patients with SCD experience mild disease with few patients requiring respiratory support (12). While these outcomes may be due to early diagnosis and treatment in an at-risk population, underlying inflammatory mechanisms in SCD may also influence COVID-19 progression.

Following SARS-CoV-2 infection, multiple cell types produce cytokines, including IL-1b, TNFα, and IL-6, which contribute to the anti-viral response and inflammation (13). Patients with SCD have chronic inflammation at baseline, characterized by leukocytosis, endothelial damage, oxidative stress, and production of pro-inflammatory cytokines. Interleukin-6 is induced during COVID-19, and there are reports of improved COVID-19 outcomes following anti-IL-6 therapy in patients with SCD (14, 15). In addition, there is particular interest in the role of anti-viral type 1 interferons (IFNα/β) produced following SARS-CoV-2 infection. IFNα/β, including IFNβ, IFNω and 12 subtypes of IFNα, were discovered in 1957 for their critical role in anti-viral immunity (16). During infection, viral nucleic acids bind pattern recognition receptors and induce IFNα/β, which in turn activate signaling via the IFNα/β receptor to produce interferon-stimulated genes (ISGs) that interfere with viral replication and promote the production of neutralizing antibodies. Independent of COVID-19, Hounkpe et al. performed a meta-analysis of gene expression studies and identified a cluster of ISGs enriched in patients with SCD (17). In addition, Hermand et al. recently reported that serum IFNα and ISGs produced by neutrophils are elevated in children with SCD, compared to healthy blood donors (18). Here, we assessed the baseline expression of a well-characterized ISG, Myxovirus Resistance Protein 1 (MxA), in adults with SCD compared to race-matched controls, and describe SARS-CoV-2 test results and COVID-19 hospitalization outcomes in one patient cohort.

## Methods and Results

**Figure 1:**
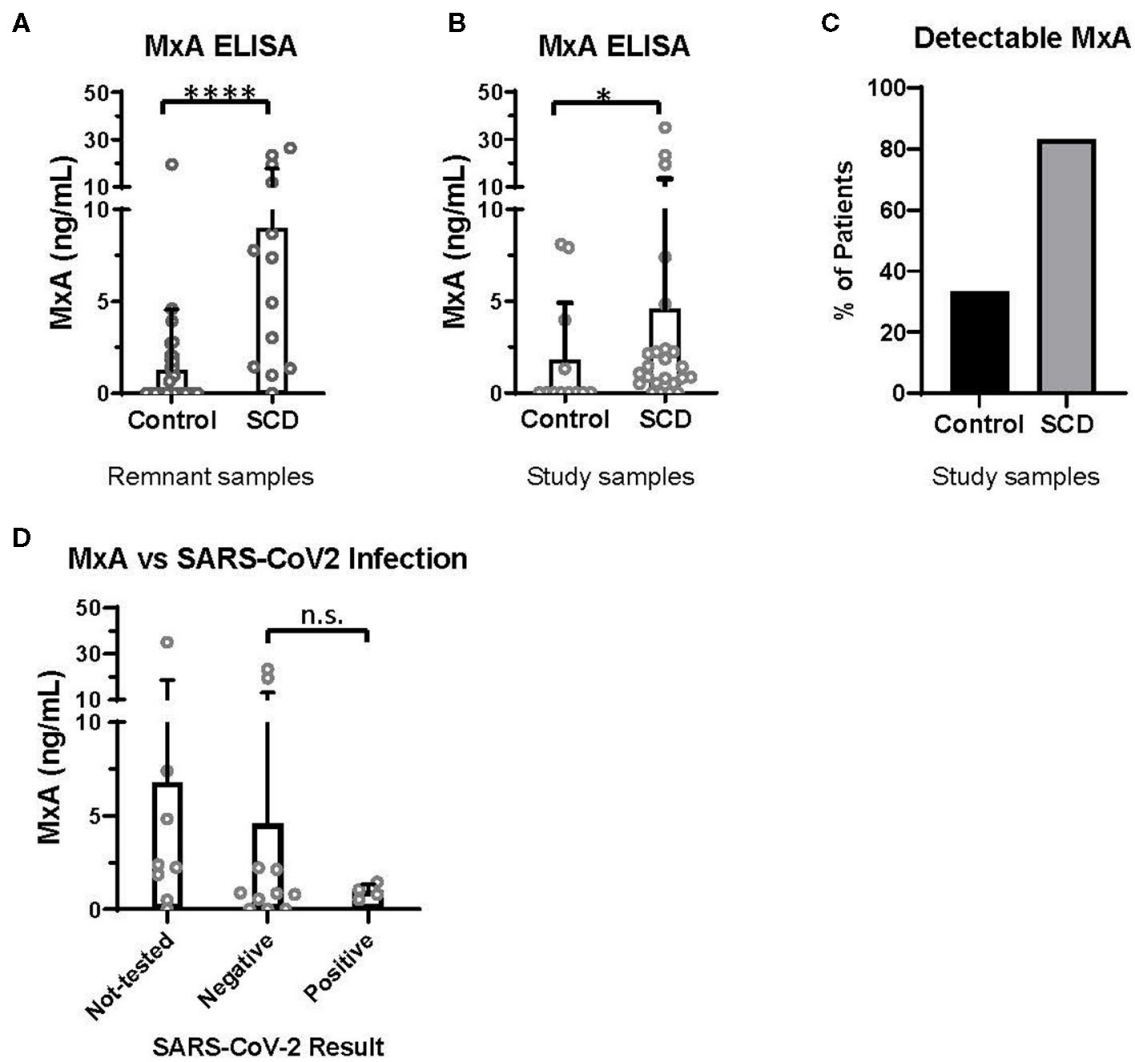
MxA expression is increased in patients with SCD, compared to controls. MxA expression in whole blood measured by MxA Protein Human ELISA (BioVendor). **(A)** MxA expression in de-identified remnant samples from patients with (*n* = 13) or without SCD (*n* = 37). **(B)** MxA expression in patients with SCD (*n* = 24) and race-matched controls (*n* = 12) summarized in Table 1. **(C)** Percentage of subjects summarized in Table 1 with detectable levels of MxA. **(D)** MxA expression of patients with SCD (*n* = 24) who were not tested or tested positive (*n* = 4) or negative (*n* = 11) for SARS-CoV-2 by PCR. n.s., not significant by Kruskal Wallis test with a Dunn's post-test. *p < 0.05, ****p < 0.0001 by Mann-Whitney *U*-test. Bars represent the mean. Circles represent values from individual patient or control samples, and error bars represent the standard deviation.

Discovered in 1962 for its role in resistance to orthomyxoviruses, including influenza, MxA is a GTPase that targets nucleocapsid proteins and inhibits viral transcription and replication (19, 20). MxA has since been reported to be a clinically applicable biomarker of IFNα/β activation (21). In the pre-COVID era, we utilized an ELISA-based assay (BioVendor, Czech Republic) to quantify MxA levels in the whole blood of patients with and without SCD, according to manufacturer instructions. Initially, 13 SCD and 37 control de-identified remnant type and screen samples were randomly selected from the blood bank. All samples were analyzed within 72 h of the blood draw. In this initial analysis, patients with SCD expressed a significantly elevated level of MxA, compared to controls (Figure 1A).

Given that these initial samples were de-identified and the health status of patients and controls was unknown, a second study comparing MxA levels in patients with SCD seen in out-patients clinics for routine care and race-matched controls was initiated. Blood was drawn from 24 patients and 12 controls in their baseline state of health. 22 patients had Hgb SS, 1 had Hgb SC, and 1 had Sβ^0^ disease (Table 1). Exclusion criteria included acute illness or crisis, pregnancy, and lack of competency to provide informed consent. The study was approved by the Yale Institutional Review Board.

**Table 1:** Demographics of subjects enrolled in second study.

		**SCD**	**Control**
Gender	Male (%)	7 (29%)	6 (50%)
	Female (%)	17 (71%)	6 (50%)
Mean age in years (Std Dev)		29.3 (9.8)	41.2 (13.7)
Hgb S disease	SS (%)	22 (92%)	
	SC (%)	1 (4%)	
	Sβ^0^ (%)	1 (4%)	

*Gender, age, and hemoglobinopathy of subjects. SCD, sickle cell disease; Std Dev, standard deviation*.

In this second study, MxA was also significantly elevated in patients with SCD compared to control subjects (Figure 1B). 83% of patients with SCD had detectable MxA, compared to 33% of controls (Figure 1C). It is noteworthy that expression of MxA, as well as IFNα and other ISGs (18), is highly variable amongst patients with SCD, which may contribute to differences in anti-viral immunity. In addition, MxA was detected in four of 12 controls, which may reflect the role of IFNα/β and MxA in other diseases, including autoimmunity and viral infections (19, 21).

Finally, we retrospectively examined SARS-CoV-2 testing results and COVID-19 severity of patients with SCD enrolled in the second study. Fifteen of the 24 patients had test results of SARS-CoV-2 PCR documented in the electronic medical record. Eleven patients had only negative test results and four patients tested positive. Three of the four with positive PCR results were admitted to the hospital: two were diagnosed with acute chest syndrome and received supplemental oxygen but neither required intubation; all patients survived. MxA levels of these patients, according to SARS-CoV-2 test results, are shown in Figure 1D. There were no significant differences in MxA levels between untested patients, patients with negative results, and patients with positive results.

## Discussion

Data presented here, in combination with prior studies (17, 18), indicate that patients with SCD express an IFNα/β gene signature. As IFNα/β and ISGs are upregulated as a result of SARS-CoV-2 infection and vaccination (22), it is worth noting that these laboratory studies were completed prior to the COVID-19 pandemic. This allowed for baseline measurement of MxA without the potential variable of SARS-CoV-2 infection or vaccination. While SARS-CoV-2 test results and COVID-19 hospitalization outcomes were examined, based on the sample size, no definitive conclusions can be drawn about baseline MxA levels and the risk of SARS-CoV-2 infection or disease severity. While it is plausible that elevated baseline anti-viral IFNα/β and ISGs, including MxA, contribute to the variable progression of COVID-19 and other viral infections in patients with SCD, it is certain that other inflammatory responses, including production of IL-1b, TNFα, and IL-6, also significantly contribute to the anti-viral response (23).

Multiple groups have investigated the role of IFNα/β in COVID-19 disease progression, independent of SCD. Utilizing mass cytometry and Nanostring technology, Hadjadj et al. performed immune profiling of 50 COVID-19 patients and observed an impaired IFNα/β response in severe and critically ill patients, compared to those with mild to moderate disease (24). Also in support of a protective role of IFNα/β, another group found inborn errors of IFNα/β immunity and an increased prevalence of autoantibodies against IFNα/β, including IFNα and IFNω, in critically ill COVID-19 patients, compared to those with mild disease or asymptomatic infection (25, 26). These results are consistent with recent reports of reduced COVID-19 associated mortality in patients treated with IFNβ (27, 28), and reduced duration of detectable virus and inflammation in a cohort treated with IFN-α2b (29).

Conversely, another group concluded that the IFNα/β response can also exacerbate deleterious COVID-19 induced inflammation. Utilizing single cell RNA sequencing, Lee et al. observed that an IFNα/β response was absent in mild disease but co-existed with Tumor Necrosis Factor α (TNFα) and IL-1b production in severe COVID-19 disease (13). However, it is unclear whether the correlation between IFNα/β and severe disease in this study is causal or responsive. In addition, Ziegler et al. demonstrated that angiotensin converting enzyme 2 (ACE2), a receptor for the SARS-CoV-2 spike protein, is an ISG expressed by multiple pulmonary cell types, suggesting that the virus may exploit the IFNα/β response to gain viral entry (30).

Although evidence supporting a protective role of IFNα/β outweighs that supporting a deleterious role in COVID-19 disease progression, it remains an area of current investigation. Ongoing clinical trials have shown conflicting results. A phase 2 trial of inhaled IFNβ showed clinical improvement (31), while data from the World Health Organization Solidarity trial indicate that IFNβ is not effective in improving mortality or other endpoints (32). Others have postulated that the timing of IFNα/β responses may impact the outcomes of the aforementioned studies (33). Early robust responses and early treatment with inhaled IFNβ may lead to reduced viral load; whereas delayed and dysregulated IFNα/β responses or IFNα/β-based treatments may exacerbate deleterious hyperinflammation at later stages of disease (34, 35).

It is noteworthy that IFNα/β has also been implicated in the pathogenesis of autoimmune diseases, including rheumatoid arthritis, myositis, Sjögren's syndrome (SS), systemic sclerosis and systemic lupus erythematosus (SLE) (36–40). Approximately two-thirds of adult patients and nearly all children with SLE express an IFNα/β gene signature (41, 42), and more than 50% of SLE-associated genetic variants have been linked to the IFNα/β pathway (43). In addition, approximately 25% of patients with SLE produce anti-IFNα/β autoantibodies, which are associated with decreased disease activity (44, 45). Due to the IFNα/β gene signature and the use of hydroxychloroquine in patients with SLE, many groups have investigated COVID-19 disease progression in patients with SLE and have reported variable results (46, 47). Unfortunately, it has been difficult to separate the effects of baseline IFNα/β activity and the use of immunosuppressive therapies, which result in an increased incidence of multiple viral infections in patients with SLE (48).

Specific mechanisms leading to IFNα/β activation during viral infection or SLE are fairly well-characterized. However, IFNα/β-inducing stimuli and pathways in SCD have not been identified. Heme-induced inflammation is one of many candidates yet to be investigated. Heme, released from hemolyzed RBCs, binds to Toll-like receptor 4 and induces NFκb-mediated production of pro-inflammatory cytokines, including IL-6, IL-12, and TNFα (49). However, its role in IFNα/β activation has not been reported, and many other ligands and pathways are worthy of investigation. Moreover, while anti-IFNα/β antibodies that prevent IFNα/β-induced inflammation have been described in patients with autoimmune polyendocrinopathy syndrome, SLE and COVID-19 (25, 50, 51), it is unclear whether they are produced by patients with SCD and whether they contribute to the variable disease progression during viral infection.

Finally, unlike in viral infection and autoimmunity, the contribution of IFNα/β activation to chronic inflammation and the numerous sequelae of SCD are unknown and warrant investigation. For example, endothelial damage is a hallmark of SCD associated with vaso-occlusive crises, including acute chest syndrome and cerebrovascular accidents, and mortality (52, 53). IFNα/β has been shown to induce endothelial damage in patients with anti-phospholipid syndrome, thrombotic microangiopathy, and SLE, and recipients of IFNα/β therapy (54–57). However, the role of IFNα/β in endothelial damage, and its associated adverse events, in SCD has not been investigated. In addition, prior studies reported that IFNα/β is a critical regulator of RBC alloimmunization in transfusion mouse models (58–61). Alloantibody production against minor RBC antigens can lead to significant hemolytic events and severely limit availability of blood products for alloimmunized patients. As patients with SCD have the highest incidence of RBC alloimmunization (62) compared with any other disease population, the role of IFNα/β in RBC alloimmunization in patients with SCD warrants further study.

In conclusion, data presented here, in combination with other studies, indicate that the majority of patients with SCD express an IFNα/β gene signature. While the impact of IFNα/β and ISGs on SARS-CoV-2 infection risk or COVID-19 disease severity remains to be fully determined, baseline IFNα/β activity may contribute to the variable disease progression reported in prior studies. Additional studies investigating mechanisms underlying IFNα/β production and clinical consequences of IFNα/β-mediated inflammation in SCD are needed. Future studies elucidating the role of IFNα/β in chronic inflammation, RBC alloimmunization, and vaso-occlusive events could lead to targeted therapies to mitigate severe sequelae in patients with SCD.

## Data Availability Statement

The original contributions presented in the study are included in the article/supplementary material, further inquiries can be directed to the corresponding author/s.

## Ethics Statement

The studies involving human participants were reviewed and approved by Yale University Institutional Review Board. Written informed consent to participate in this study was provided by the participants or the participants' legal guardian/next of kin.

## Author Contributions

Project design and data analysis were completed by EM, DO-D, JEH, and DRG. Experiments were completed by DRG, EM, and RB-M. DRG wrote the initial draft of the manuscript. All authors contributed to edits of the manuscript.

## Conflict of Interest

The authors declare that the research was conducted in the absence of any commercial or financial relationships that could be construed as a potential conflict of interest.
